# The wheat Lr34 multipathogen resistance gene confers resistance to anthracnose and rust in sorghum

**DOI:** 10.1111/pbi.12723

**Published:** 2017-04-20

**Authors:** Wendelin Schnippenkoetter, Clive Lo, Guoquan Liu, Katherine Dibley, Wai Lung Chan, Jodie White, Ricky Milne, Alexander Zwart, Eunjung Kwong, Beat Keller, Ian Godwin, Simon G. Krattinger, Evans Lagudah

**Affiliations:** ^1^ CSIRO Agriculture and Food Canberra ACT Australia; ^2^ School of Biological Sciences The University of Hong Kong Hong Kong China; ^3^ School of Agriculture and Food Sciences The University of Queensland St Lucia QLD Australia; ^4^ Centre for Crop Health University of Southern Queensland Toowoomba QLD Australia; ^5^ CSIRO Data61 Canberra ACT Australia; ^6^ Department of Plant and Microbial Biology University of Zurich Zurich Switzerland; ^7^ School of Life and Environmental Sciences University of Sydney Sydney NSW Australia

**Keywords:** multiple disease resistance, Lr34, rust, anthracnose, flavonoid phytoalexin

## Abstract

The ability of the wheat Lr34 multipathogen resistance gene (*Lr34res*) to function across a wide taxonomic boundary was investigated in transgenic *Sorghum bicolor*. Increased resistance to sorghum rust and anthracnose disease symptoms following infection with the biotrophic pathogen *Puccinia purpurea* and the hemibiotroph *Colletotrichum sublineolum*, respectively, occurred in transgenic plants expressing the *Lr34res* ABC transporter. Transgenic sorghum lines that highly expressed the wheat *Lr34res* gene exhibited immunity to sorghum rust compared to the low‐expressing single copy *Lr34res* genotype that conferred partial resistance. Pathogen‐induced pigmentation mediated by flavonoid phytoalexins was evident on transgenic sorghum leaves following *P. purpurea* infection within 24–72 h, which paralleled *Lr34res* gene expression. Elevated expression of *flavone synthase II*,* flavanone 4‐reductase* and *dihydroflavonol reductase* genes which control the biosynthesis of flavonoid phytoalexins characterized the highly expressing *Lr34res* transgenic lines 24‐h post‐inoculation with *P. purpurea*. Metabolite analysis of mesocotyls infected with *C. sublineolum* showed increased levels of 3‐deoxyanthocyanidin metabolites were associated with *Lr34res* expression, concomitant with reduced symptoms of anthracnose.

## Introduction

Sorghum (*Sorghum bicolor*) is ranked as the fifth most commonly cultivated cereal in the world (FAOSTAT, 2016). Some of its useful attributes are tolerance to dry environments, high sugar content, high yields of forage biomass per unit of cultivated area and as a rich source of distinct phytochemicals such as dhurrin, sorgoleone and 3‐deoxyanthocyanidins. While sorghum provides a useful resource for industrial purposes, for example the generation of ethanol, fibre and paper, its primary use is still for feed and food especially in the semi‐arid tropics. Protecting yield losses from diseases such as anthracnose (*Colletotrichum sublineolum*) and rust (*Puccinia purpurea*), which can be variable in different agro‐ecological regions continues to be a goal of sorghum improvement. Grain size and yield losses over 50% have been reported with anthracnose epidemics (Thakur and Mathur, 2000). Rust is particularly problematic in late‐sown crops (White *et al*., 2012) with yield losses up to 65% (Bandyopadhyay, 2000).

Genetic solutions to protect crop plants against pathogens are often preferable to the use of agrochemicals. Numerous studies on plant‐microbe interaction have led to an increased understanding of the molecular basis of the plant defense system, depicted by multiple layers of the plants ability to resist pathogen proliferation (Dangl *et al*., 2013). While most resistance genes tend to be short‐lived, certain forms of plant defense genes provide more durable resistance. Studies in wheat with defined races of *Puccinia* (rust) and *Blumeria* (mildew) pathogen species have resulted in over 220 catalogued resistance genes, most of which individually provide resistance to a few races of a specific pathogen (McIntosh *et al*., 2013). However, a small number (e.g. *Lr34*,* Lr46* and *Lr67*) have been identified that confer adult plant, broad spectrum partial resistance to multiple pathogen species. Most notable among the latter class of resistance genes is the *Lr34* multipathogen resistance gene (Dyck and Samborski, 1979; McIntosh, 1992; Singh, 1992; Spielmeyer *et al*., 2005), which has been successfully deployed in wheat cultivation and provided durable field resistance to rust pathogens for over 100 years (Kolmer *et al*., 2008). Significantly, the multipathogen resistance conferred by *Lr34* was not due to a cluster of resistance genes, but rather by a single gene encoding an ABC transporter (Krattinger *et al*., 2009; Risk *et al*., 2012). *Lr34* also differs from the other cloned multipathogen resistance gene *Lr67*, which encodes a sugar transporter from the STP13 lineage of monosaccharide transporters (Moore *et al*., 2015). The Lr34 resistance allele (*Lr34res*) differs from the susceptible or wild‐type allele, *Lr34sus*, by changes to two amino acids; loss of a phenylalanine residue (F546) and a tyrosine to histidine substitution (Y634H) located in two separate, predicted transmembrane helices. The mechanism of disease resistance conferred by *Lr34res* remains unknown as does the substrate(s) it may translocate. Nonetheless, transfer of *Lr34res* as a transgene to other crops such as barley and rice has demonstrated its capability in conferring resistance against other pathogens that are unadapted to wheat (Krattinger *et al*., 2016; Risk *et al*., 2013).

While *Lr34res* in wheat confers partial adult plant resistance to predominantly biotrophic pathogens, the observation of *Lr34res* efficacy in transgenic rice against the hemibiotroph, *Magnaporthe grisea* (causal agent of rice blast; Krattinger *et al*., 2016), led us in this study to examine the effect of *Lr34res* against the hemibiotroph pathosystem of anthracnose in sorghum. Secondly, we also note that while *Lr34res* effectiveness has been demonstrated against *Puccinia* species (*P. hordeii*,* P. striiformis*,* P. graminis*, and *P. triticina*) adapted to cool season crops, it remains unknown as to its function against *Puccinia* species that are virulent on warm season crops such as *P. purpurea* in sorghum. Thirdly, we take advantage of the pathogen‐inducible visible pigmentation changes resulting from the synthesis of a unique class of flavonoid phytoalexins in sorghum, in furthering our understanding of potential signalling pathways triggered by *Lr34res*.

## Results

### Transgenic *Lr34res* expression confers resistance to sorghum rust (*Puccinia purpurea*) infection

We introduced the complete wheat genomic sequence of *Lr34res*, encompassing 2.4 kb of native promoter and 1.5 kb native terminator sequence, by stable transformation in the genetic background of sorghum cultivar (cv.) Tx430. Four independent T0 transformants with the full‐length *Lr34res* were obtained, of which three independent genotypes were fertile (Lr34‐2, Lr34‐5 and Lr34‐6). Subsequent genomic and phenotypic analyses at T1–T3 generations were carried out on these three independent transgenic lines. Genomic blot analysis showed that line Lr34‐2 carried a single copy of the *Lr34res* gene, whereas multiple copies were detected in lines Lr34‐5 (three copies) and Lr34‐6 (seven copies; Figure S1). Analysis of individual T3 plants from the multicopy *Lr34res* events showed identical genomic hybridization patterns to the Lr34 probe that were unique to either Lr34‐5 or Lr34‐6 T3 progeny. This suggests that multicopy events in Lr34‐5 and Lr34‐6 were inserted at single sites or in close proximity and consequently the absence of segregation of the transgenes.

**Figure 1:**
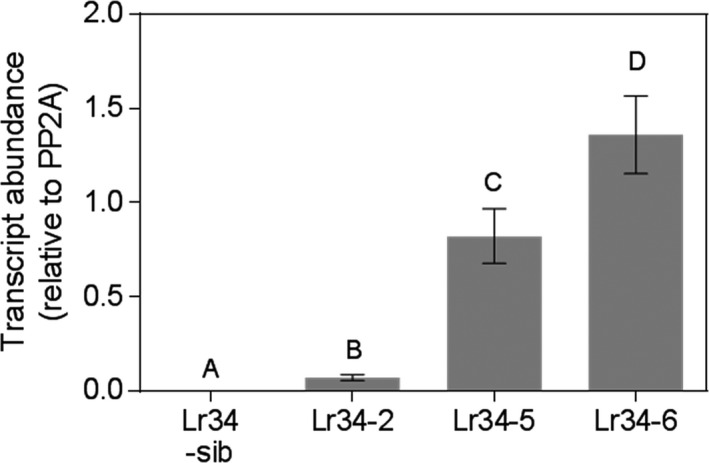
*Lr34res* expression levels in adult plants of transgenic sorghum. Lr34‐sib negative line, Lr34‐2 single copy line, Lr34‐5 3 copy line, Lr34‐6 7 copy line. Data shown as mean ± SE from three biological replicates.

Given that Lr34 transgenic barley and rice plants exhibited the leaf tip necrosis (Ltn) phenotype at an earlier developmental stage than wheat (Krattinger *et al*., 2016; Risk *et al*., 2013), we monitored the transgenic sorghum plants for similar morphological changes. Phenotypically, the control plants (sib lines without the transgene) and the *Lr34res* single copy line, Lr34‐2, were very similar at the seedling stage until the onset of booting. Yellowing leaf margins and leaf tips, typical of the Ltn phenotype, occurred earlier in Lr34‐2 compared with adult plants lacking the transgene. In contrast, Lr34‐5 and Lr34‐6 lines showed a progressive development of a blotchy bronze/purple leaf coloration in adult plants from about the penultimate leaf development stage onwards (Figure S2). The penultimate leaves of adult plants had high *Lr34res* transcript levels in lines Lr34‐5 and Lr34‐6, which was 8–13 fold higher than that detected in the single copy Lr34‐2 genotype (Figure 1). Thus, the strong leaf coloration phenotypes correlated with *Lr34res* expression.

**Figure 2:**
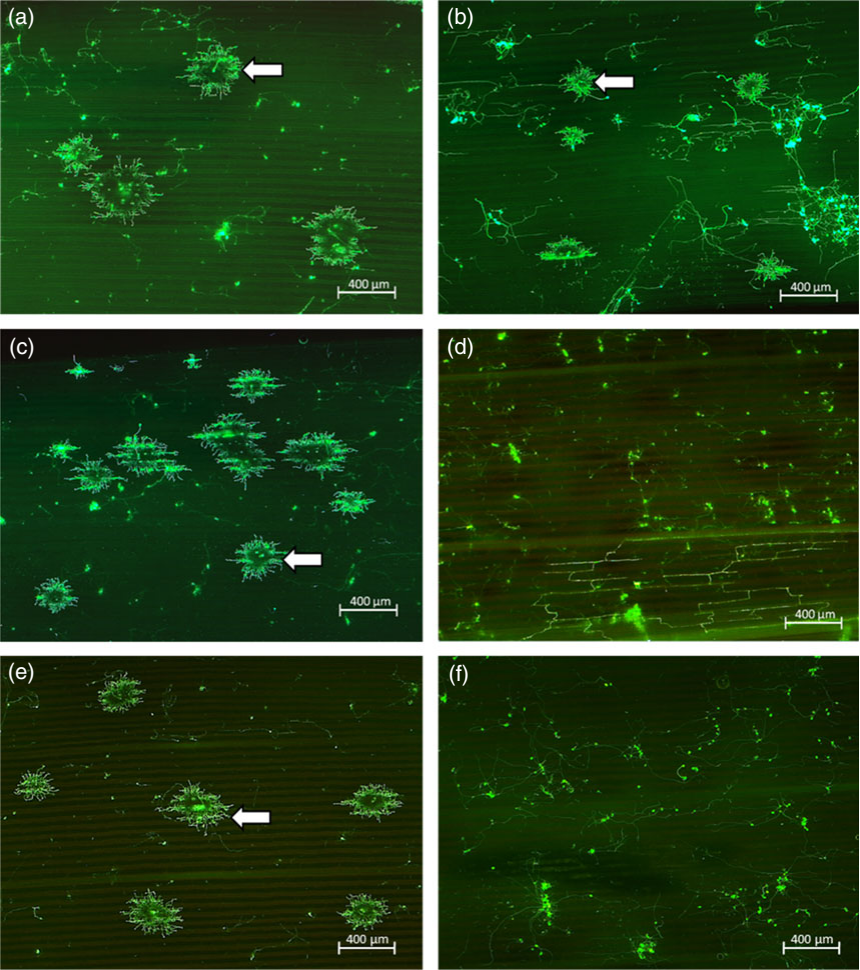
Micrographs of sorghum rust development following WGA‐FITC staining at 7 days post‐inoculation of fifth leaves. (a) Wild‐type Sorghum cultivar Tx430. (b) Sorghum landrace. (c) and (e) Segregate Sib lines Lr34‐2 and Lr34‐5 respectively not harbouring *Lr34res* gene – Infection sites (arrows) developing from germinated rust spores on leaf surfaces. (d) and (f) Lr34‐2 and Lr34‐5 transgenic sorghum respectively–germinated spores and hyphae present on leaf surface but with no infection sites. Micrographs of Lr34‐6 negative sib and transgenic lines yielded similar results to that of the negative sib and transgenic lines of Lr34‐2 and Lr34‐5, respectively.

**Figure 3:**
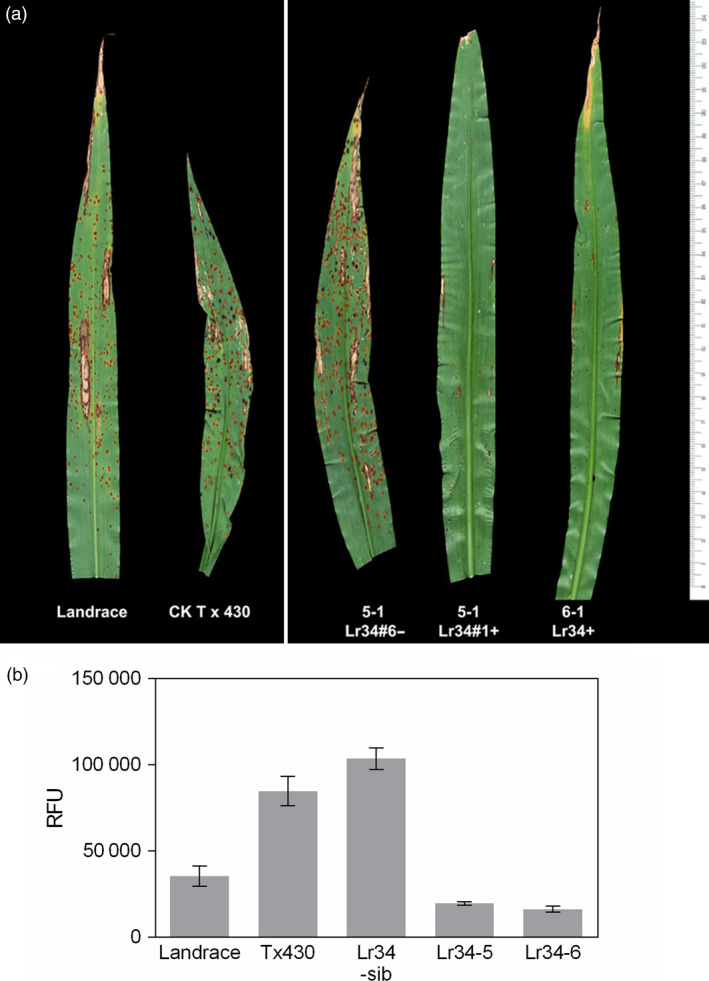
(a) *P. purpurea* pustule development on control and transgenic sorghum leaves at 14 dpi. (b) Quantification of fungal biomass on corresponding plants in (a). Data shown as mean ± SD.

Sorghum rust pathogenesis on plants infected by *P. purpurea* urediospores at the 5‐leaf stage was analysed microscopically at 7 days post‐inoculation (dpi) and for sporulation at 12–14 dpi. Microscopic analysis of wheat germ agglutinin‐fluorescein isothiocyanate (WGA‐FITC) binding to fungal cell walls revealed extensive hyphal development in control plants and sib lines without the *Lr34res* transgene (Figure 2a‐c, e). In contrast, hyphal growth from infection sites in all transgenic *Lr34res* lines was restricted (Figure 2d, f). Macroscopically, spores from uredinia developed on all the non*Lr34res* plants, whereas no sporulation was detected on *Lr34res* transgenic genotypes (Figures 3a and S3). Further quantification of the sorghum rust fungal biomass on transgenic plants showed the presence of the *Lr34res* transgene reduced fungal colonization by 75%–80% (Figure 3b). Interestingly, by 28–30 dpi, uredinia had developed on the Lr34‐2 transgenic line, albeit at low frequency compared to sib lines lacking *Lr34res* (Figure S4). Estimation of fungal biomass at this late period showed approximately a 25% reduction in fungal colonization, which is indicative of the slow rusting phenotype that typifies the partial resistance often seen with *Lr34res* in wheat. In contrast, no sporulation was detected on Lr34‐5 and Lr34‐6 genotypes, even at this late stage.

From previous analysis of the *Sorghum bicolor* genome, two adjacent Lr34 orthologs, Sb01g016770 and Sb01g016775, were considered to have arisen by gene duplication, of which Sb01g016770 was deduced to be a pseudogene (Krattinger *et al*., 2013). Sb01g016770 and Sb01g016775 share 71% and 75% identity respectively with the protein sequence of LR34. Of the two critical amino acids that distinguish LR34RES from LR34SUS, Sb01g016775 shared the same phenylalanine and tyrosine residues found in the wild‐type variant of LR34SUS. We investigated by site directed mutagenesis whether changes to Sb01g016775 involving the two critical amino acids to a modified Sb01g016775 with a deleted phenylalanine (∆F525) and tyrosine to histidine (Y613H) was capable of conferring resistance to sorghum rust as observed with *Lr34res*. Five independent stable transgenic lines of Sb01g016775^−∆F525, Y613H^ were generated, all of which expressed transcripts carrying the modified gene. Plants infected with *P. purpurea* developed similar levels of sporulation as control plants or sib lines by 14 dpi and failed to exhibit the resistance phenotype that accompanied the introduction of the wheat *Lr34res* in sorghum (Figure S5).

### Pathogen‐induced leaf pigmentation, expression of genes involved in the flavonoid phytoalexin synthesis pathway and metabolite analysis

**Figure 4:**
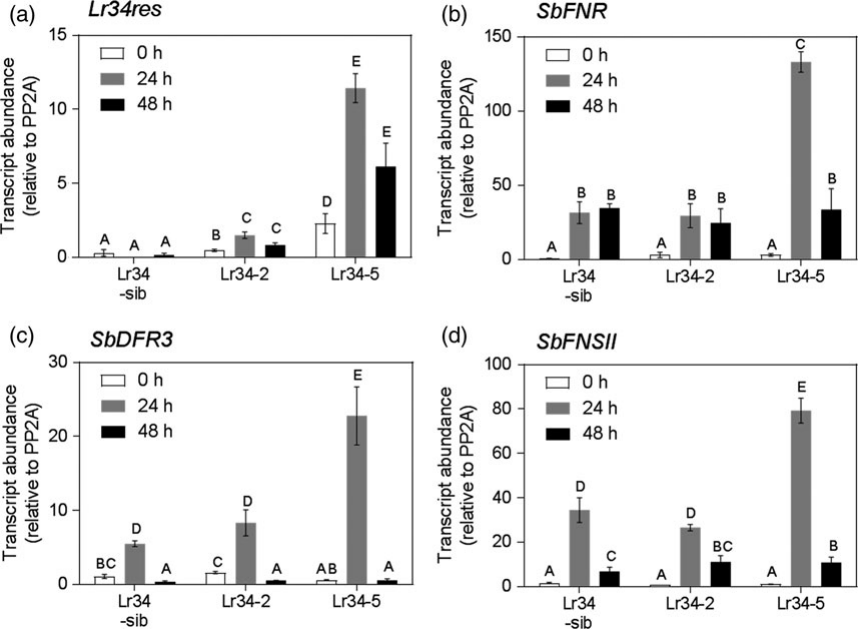
Comparative pathogen‐induced gene expression pre‐and post‐inoculation with *P. purpurea*. (a) *Lr34res*. (b) *SbFNR*. (c) *SbDFR3*. (d) *SbFNSII*. Data shown as mean ± SE from three biological replicates.

Within the first 2 days following *P. purpurea* inoculation, reddish brown pigmented spots were observed on leaves of control and transgenic plants. Leaf area coverage and size of pigmented spots were larger in transgenic plants when compared to non‐transgenic sibs and the control genotype (Figure S6). Furthermore, the *Lr34res* multicopy genotypes Lr34‐5 and Lr34‐6 consistently exhibited more pigmented areas than the single copy Lr34‐2 transgenic line. To test whether the magnitude of the pathogen‐induced pigmentation was associated with *Lr34* expression, transcript levels of *Lr34res* were quantified over a 48‐h period post‐inoculation. An increase in the *Lr34res* transcript occurred within 24‐h post‐inoculation (hpi) and declined by 48 hpi (Figure 4). More than threefold increased expression occurred in Lr34‐5 at 24 hpi compared to Lr34‐2, which parallels the extent of pigmentation noted on the leaves. We also examined the expression levels of the *S. bicolor* orthologous *Lr34* gene, Sb01g016775 under mock and rust inoculation in comparison with the introduced *Lr34res* transgene. Interestingly, Sb01g016775 expression was negatively responsive to *P. purpurea* inoculation in contrast to the pathogen responsiveness of the wheat *Lr34res* demonstrated through increased expression (Figure S7).

**Figure 5:**
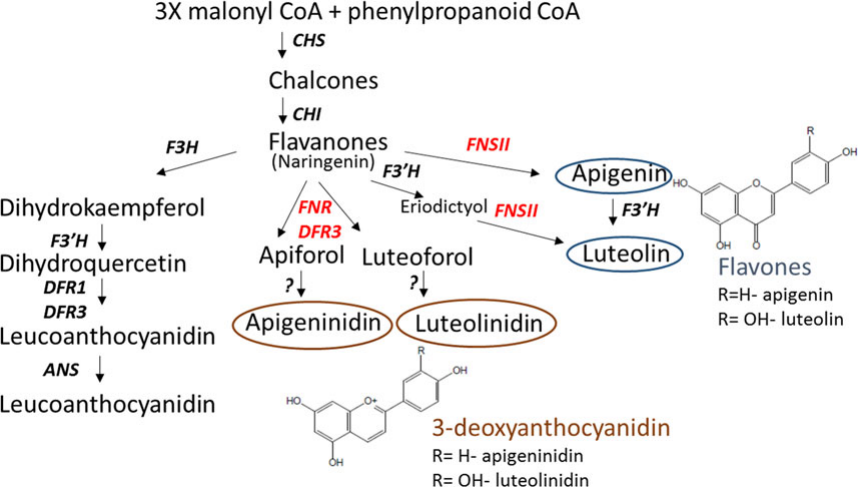
Flavonoid phytoalexin and anthocyanidin biosynthetic pathway (Kawahigashi *et al*., 2016; Liu *et al*., 2010). Genes highlighted in red and products circled were quantified in this study.

Pathogen‐inducible synthesis of flavanone derived metabolites, some of which have been implicated in plant defense, has previously been described in sorghum (Lo *et al*., 1996, 1999; Nicholson *et al*., 1987). Analysis of expression levels of key enzymes involved in 3‐deoxyanthocyanidin and flavone biosynthesis (Figures 4b–d, 5) revealed similar trends to effects of the *Lr34res* transgene. Enzymatic steps encoded by *SbFNSII* (flavone synthase II, a cytochrome P450 pathogen‐inducible gene), *SbFNR* (flavanone 4‐reductase) and *SbDFR3* (dihydroflavonol reductase) were elevated in gene expression at 24 hpi and declined at 48 hpi (Figure 4b–d). The high expressing *Lr34res* lines, typified by Lr34‐5 genotype, exhibited over 15‐, 75‐ and 140‐fold increases in expression of *SbDFR3*,* SbFNSII* and *SbFNR*, respectively, at the peak period of 24 hpi. By contrast the control sib line showed 5‐, 15‐ and 20‐fold increases for *SbDFR3*,* SbFNSII* and *SbFNR*, respectively. Approximately an eightfold elevation of *SbDFR3* was detected in the low *Lr34res* expressing genotype, Lr34‐2, over the same period, whereas *SbFNSII* and *SbFNR* showed similar quantitative changes in the control line and Lr34‐2 (Figure 4b–d). Taken together, the early induction of this group of genes which form part of the pathway in converting naringenin flavanones to 3‐deoxyanthocyanidin and flavone biosynthesis (Figure 5) is enhanced by the introduction of the wheat *Lr34res* gene upon pathogen infection.

**Figure 6:**
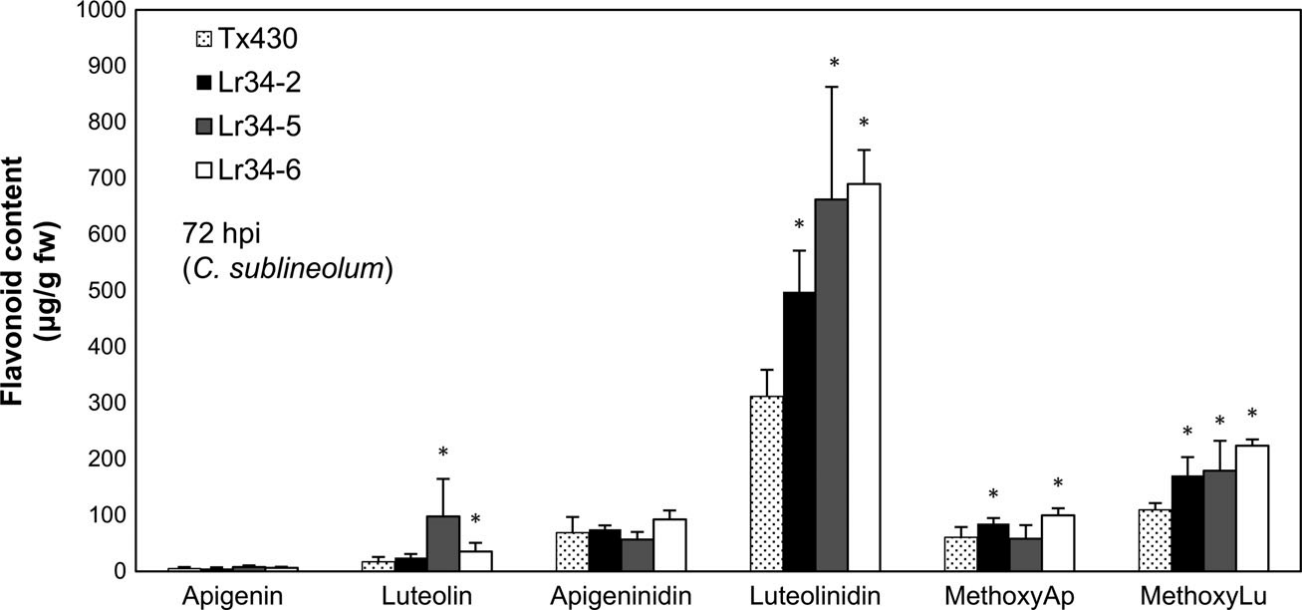
Metabolite analysis of 3‐deoxyanthocyanidn and flavone phytoalexins measured in sorghum mesocotyls 72‐h post‐inoculation with *C. sublineolum*. Data shown as mean ± SD from three biological replicates. *P < 0.05 (*t*‐test).

We further investigated the production of metabolites that belong to the 3‐deoxyanthocyanidin class upon infection using the well‐studied *C. sublineolum*‐sorghum pathogen‐host interaction. Pathogen‐induced formation of purple pigments has attributed this colour change to structurally related compounds, 3‐deoxyanthocyanidins (luteolinidin and apigeninidin; Dykes and Rooney, 2006). These compounds accumulate within inclusions in the epidermal cells as a defense response to pathogen attack (Snyder and Nicholson, 1990; Snyder *et al*., 1991). As part of the metabolite analysis we also included the flavone aglycones, luteolin and apigenin, that have also been implicated to differentially accumulate as sorghum phytoalexins in response to pathogen infection (Du *et al*., 2010). Metabolite analysis was conducted on elongated mesocotyls inoculated with *C. sublineolum* at 48, 72 and 96 hpi. Significantly enhanced levels of luteolinidin were detected in genotypes carrying *Lr34res* at 72 hpi (Figure 6). Differences in metabolite accumulation were not as significant at the other two time points (Figure S8). At 48 hpi, metabolite amounts were still rather low and by 96 hpi, the 3‐deoxyanthocyanidins started to be degraded. Methoxyluteolinidin and methoxyapigenidin levels were also significantly higher in most transgenic lines at 72 hpi, but their levels were not as high as luteolinidin. As expected, the flavones (luteolin and apigenin) accumulated at considerably lower levels than luteolinidin, while some elevation of luteolin levels was detected in the high expressing *Lr34res* genotypes, L34‐5 and L34‐6 (Figure 6).

### Transgenic *Lr34res* expression confers resistance to sorghum anthracnose (*C. sublineolum*)

**Figure 7:**
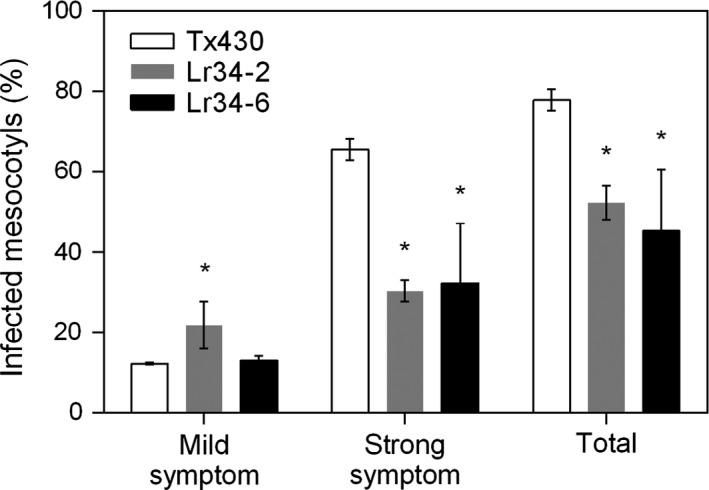
Spectrum of infection following *C. sublinoleum* inoculation of mesocotyls of control and transgenic sorghum lines. Data shown as mean ± SD from three biological replicates. *: Significantly different from the corresponding values in Tx430 (*t*‐test, *P*‐value <0.05); Total = mild + strong

In addition to the metabolite analysis, we investigated the effect of the *Lr34res* transgene on disease symptoms caused by infection with *C. sublineolum*. Necrotic lesion phenotypes on elongated mesocotyls were examined at 7 dpi. Mild symptoms were characterized by single localized lesions, whereas strong symptoms were associated with multiple or complete lesions along the entire length of the mesocotyl (Figure S9). Strong anthracnose symptoms developed on 65% of the control lines compared with 30% in genotypes carrying the *Lr34res* transgene (Figure 7). Analysis of the total symptoms showed approximately 33% and 26% reduction in disease severity associated with the high and low expressing Lr34res lines, respectively (Figure 7). However, mild symptoms occurred twice as much in the single copy transgenic line compared with the higher *Lr34res* expressing genotype.

### Effect of the *Lr34res* transgene on plant vigour

**Figure 8:**
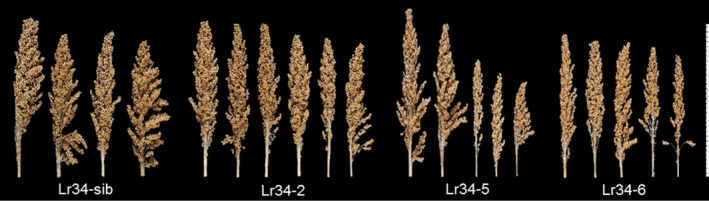
Effects of the *Lr34res* transgene on panicle morphology. T2 generation primary panicles grouped from respective sorghum Lr34res transgenic lines.

**Figure 9:**
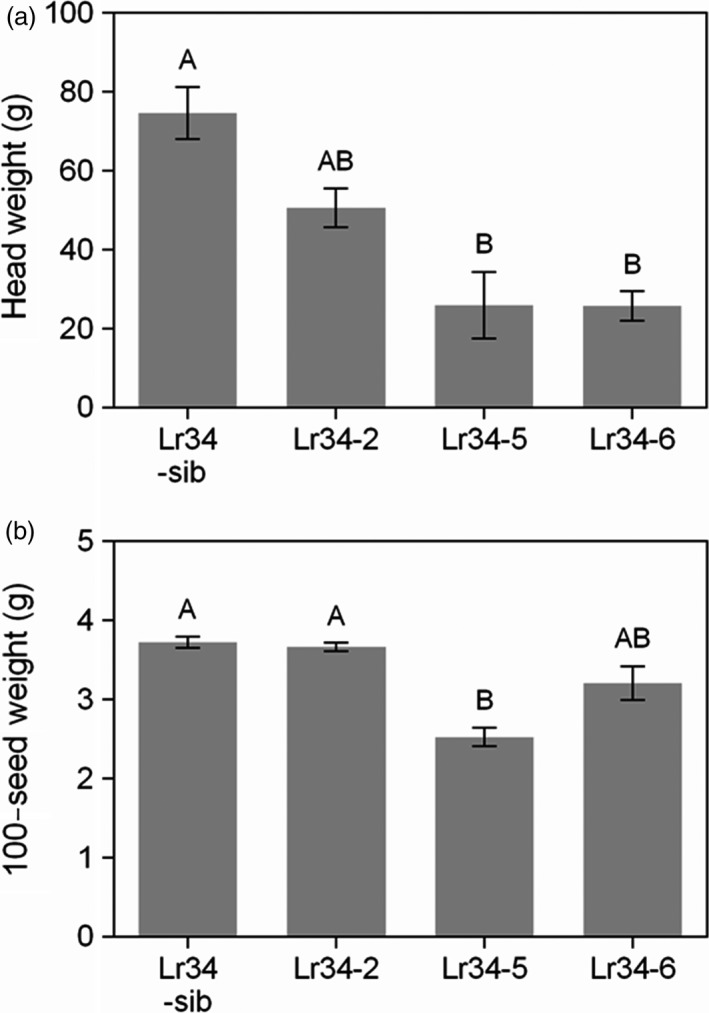
Effects of the *Lr34res* transgene on panicle yield. (a) Panicle weights and (b) 100‐seed weight of transgenic sorghum lines. Data shown as mean ± SE from 4 to 6 biological replicates.

As a general observation, no differences in plant growth vigour were noticed among the control sib lines and *Lr34res* transgenic lines during the seedling stage and even after the 5‐leaf stage when rust inoculations were conducted. However, as the plants approached booting, it was evident that the high expressing *Lr34res* genotypes (Lr34‐5 and Lr34‐6) were less vigorous in growth compared to the single copy line (Lr34‐2) and sib lines lacking the transgene. To quantify the growth effects and subsequent effect on reproductive development and yield, aspects of panicle morphology and yield were measured. Panicle size tended to be smaller in genotypes with increased *Lr34res* gene copy number and expression (Figure 8). The mean panicle weight declined by 33% and 67%, respectively, in the single copy and multicopy Lr34res lines, respectively, as compared with the negative sib lacking *Lr34res* (Figure 9a). The mean peduncle diameter in comparison with the negative sib lacking *Lr34res* (measured immediately below the node of the basal rachis), was reduced by 1.0 mm in the single copy *Lr34res* line and 3.2 mm in the multicopy Lr34res genotypes (Figure S10). The grain yield component of 100‐seed weight remained unchanged between the control sib and the Lr34‐2 line, whereas a reduction of 0.5–1.2 g occurred in the multicopy *Lr34res* lines (Figure 9).

## Discussion

We demonstrate in this study that the ABC transporter encoded by the wheat *Lr34res* gene functions in sorghum and confers resistance to sorghum‐adapted rust and anthracnose causing pathogens, while *Lr34res*‐mediated resistance to rust caused by *Puccinia* species has previously been confined to species in the Triticeae (Dyck and Samborski, 1982; Rinaldo *et al*., 2016; Risk *et al*., 2013), our findings together with the recently reported observations in maize (Sucher *et al*., 2016) extends the efficacy of *Lr34res* to the warm season adapted *Puccinia* species with pathogenesis on Andropogoneae taxa. The successful incorporation of *Lr34res*‐mediated resistance into sorghum suggests that the necessary components required for biosynthesis of the Lr34 putative substrate, and proteins involved in signalling and defense response, are also present in sorghum. This finding is of importance as it opens alternate avenues to explore the genetic dissection of *Lr34res*‐mediated resistance. Indeed, the well characterized features of pathogen‐induced pigmentation in sorghum, and associated flavonoid phytoalexin biosynthesis defense response, are avenues that were further investigated in this study.

While most of the pathogen‐inducible pigments formed in sorghum have been reported with the *C. sublineolum* and *Cochliobolus heterostrophus* pathosystems, we show in this study that *P. purpurea* infection triggers similar phenotypes as part of the early host response in the sorghum cv. Tx430. Such visible phenotypes in wheat plants with *Lr34res* or other rust resistance genes at 24–72 hpi are yet to be reported. The correlation of *Lr34res* expression and strength of pathogen‐induced pigmentation suggests that the *Lr34* transgene interacts with the signalling response, triggering pigmentation. Because the pigments responsible for the pathogen‐induced colour changes formed in sorghum are derived predominantly from the 3‐deoxyanthocyanidin flavonoids luteolinidin and apigeninidin, which accumulate as a site‐specific response to fungal infection (Nicholson *et al*., 1987; Snyder and Nicholson, 1990), it is conceivable that the presence of the *Lr34res* transgene may contribute to their elevated accumulation in infected plants. Accumulation of the 3‐deoxyanthocyanidins, in particular luteolinidin, occurs much faster in infected cells of resistant genotypes than susceptible genotypes, implicating early phytoalexin accumulation in preventing disease spread by restricting proliferation of fungal hyphae (Basavaraju *et al*., 2009; Poloni and Schirawski, 2014; Wharton and Julian, 1996). In infected cells, the 3‐deoxyanthocyanidins migrate to the site of attempted penetration dependent on nuclear migration, cytoplasmic streaming and intracellular pH to provide an environment for inclusion trafficking and release of the phytoalexins (Nielsen *et al*., 2004). Exactly how *Lr34res* fits into this transport processes remains to be defined. Notwithstanding, it is noteworthy that at 24 hpi by *P. purpurea*, the strong expressing *Lr34res* transgenic lines exhibited higher expression levels for *FNR*,* DFR3* and *FNSII* genes that form part of the flavonoid phytoalexin biosynthesis pathway. Interestingly, the introduction of *Lr34res* into barley also resulted in constitutive up‐regulation of genes involved in the flavonoid pathway and in the biosynthesis of barley defense compounds, such as *anthranilate synthase*,* anthranilate N‐benzoyltransferase*,* agmatine coumaroyl transferase* and *flavonoid 7‐O‐methyl transferase* (Chauhan *et al*., 2015).

*Lr34res* in hexaploid wheat typically provides partial resistance to rusts and mildew in adult plants, although under low‐temperature conditions (>10°C) seedling resistance can be detected. In the current study, we show that *Lr34res* functions in seedlings and obviates the need for low‐temperature induction in transgenic sorghum to provide resistance to sorghum rust and anthracnose. This observation corroborates seedling resistance by *Lr34res* against other pathogens reported in barley, rice, maize and durum wheat. The obvious difference in sorghum to other species being the highly expressed pathogen‐inducible purple coloration due to phytoalexin production. Expression levels of *Lr34res* in hexaploid wheat seedlings are elevated under low temperatures in rust‐infected plants (Rinaldo *et al*., 2016; Risk *et al*., 2013) and may account for the low temperature‐induced resistance. In transgenic sorghum, barley, rice, maize and durum wheat seedlings, it is likely that the expression level of the *Lr34res* transgene upon pathogen infection reaches a threshold level that is sufficient to trigger resistance by curbing pathogen proliferation. The leaf tip necrosis/early senescence phenotype of lines carrying *Lr34res* suggests a common pathway confers resistance.

Orthologues of the wheat *Lr34* gene are present in the sorghum and rice genomes. Targeted changes to the two amino acids in the sorghum LR34 orthologue to mimic the wheat LR34RES failed to generate a resistance phenotype similar to previous studies with the rice orthologue (Krattinger *et al*., 2016). Given the amino acid sequence identity of 75% between the wheat and sorghum orthologues of the LR34 ABC transporter, it is possible that other regions of the LR34RES absent in sorghum are required for resistance function. Additionally, the *Lr34res* in transgenic sorghum is up‐regulated upon pathogen infection, but the corresponding sorghum *Lr34* orthologue showed a weak negative response to pathogen challenge, which may also account for the lack of resistance phenotype associated with the modified sorghum LR34 orthologue. Thus any attempts at further modifications to the sorghum LR34 orthologue towards engineering resistance will likely require changes beyond the coding region to include pathogen responsive regulatory sequences.

Overexpression of *Lr34res* typified by the multicopy transgenic Lr34‐5 and Lr34‐6 genotypes in this study results in plants with reduced yield components, despite exhibiting immunity against *P. purpurea*. Conversely, the single copy low‐expressing line (Lr34‐2) had similar growth vigour as the non‐transgenic or sib line control plants and less detrimental effects on reproductive yield. As Lr34‐2 showed no rust symptoms 2 weeks post‐infection and reduced rust sporulation after 4 weeks, indicative of the characteristic slow rusting response of the *Lr34res*, and it holds promise for the use of *Lr34* as a transgene for sorghum improvement. It may also be useful to explore high expression of *Lr34res* for plant immunity to various pathogens using pathogen‐inducible promoters in an attempt to overcome detrimental reproductive yield effects associated with constitutive overexpression in adult plants. Our findings in sorghum that *Lr34res* confers resistance to sorghum rust and anthracnose demonstrates that the multipathogen resistance of the wheat *Lr34* gene extends to biotrophic and hemibiotrophic adapted pathogens across the Triticeae, Oryzeae and Andropogoneae taxa.

## Experimental procedures

### Production of transgenic Lr34 sorghum

The genomic construct of *Lr34res* under the native promoter and terminator sequences was cloned into plasmid pWGEM‐NZf as previously described (Risk *et al*., 2012) and subsequently transformed into the sorghum inbred line Tx430 via microprojectile‐mediated transformation (Liu and Godwin 2012). The presence of the transgene in T0 plants was initially assessed by PCR with *Lr34res*‐specific primers (Lagudah *et al*., 2009) and subsequently by genomic blots probed with the Lr34 3′UTR DNA fragment. A genomic construct containing the sorghum Lr34 ortholog (Sb01g016775) was generated from an EagI 16.3 kb DNA fragment from a sorghum BAC clone (CUGI BAC#156N20) subcloned into pWGEM‐NZf. Site directed mutagenesis using primers Sb2Quickchange 1F/1R (1F‐TGGGAGCATTATATTTTTCCATCATCATTATGCTAAATGGCATACC/1R‐ GGTATGCCATTTAGCATAATGATGATGGAAAAATATAATGCTCCCA) and Sb2Quickchange 2F/R (2F‐ CATCAATCAGTAATGGCGTTCCATCGATTTGTCGCTTCTTATG/2R‐ CATAAGAAGCGACAAATCGATGGAACGCCATTACTGATTGATG) was used to generate the derived subclone Sb01g016775^−∆F525, Y613H^ as per protocols in Krattinger *et al*. (2016). Transgenic plants with the genomic construct of Sb01g016775^−∆F525, Y613H^ with its native promoter and terminator sequences were also generated by microprojectile bombardment.

### Identification of transformants and Lr34 copy number

Leaf samples (2–3 g) from T1 and T3 plants were ground in liquid nitrogen using pestle and mortars and sand. Frozen leaf material was transferred into 3 mL CTAB extraction buffer (100 mm Tris‐HCl pH 8.0, 20 mm EDTA, 1.4M NaCl, 0.5% Na_2_S_2_S_5_, 2% CTAB, and 1% β‐mercaptoethanol) and processed for DNA isolation in accordance with Collins *et al*. (1998). About 12 μg of each gDNA sample was subjected to NotI and EcoRV restriction endonuclease digestions to ascertain the presence of full‐length *Lr34* gene constructs and the copy number of *Lr34* in transgenic sorghum lines, respectively. Digested gDNA samples were loaded on 1% agarose gels and run at 60V (at approx. 50 mA) for 18–20 h, capillary transferred onto Hybond‐N+^®^ filter using 20xSSC buffer and UV cross‐linked. Filter was subjected to 5‐h prehybridization in 30 mL prehybridization solution containing salmon sperm DNA at 65°C in a hybridization tube. Subsequent hybridization incorporating 50 ng of probe DNA (^32^P‐labelled Lr34‐3′UTR probe from the amplicon generated using ABCTEX1314F‐ CAGAACACCTACAGAAGAATATC and ABCR9‐ GGCAAGTAGCTATATCTGTAAC) was performed according to Lagudah *et al*. (1991).

### Sorghum rust inoculation

Sorghum seed was germinated and grown in pots at 20°C in a glasshouse or in a growth chamber. Plants at the 5–7 leaf stage were placed in closed inoculation chambers and allowed to acclimatize for 18–24 h. *Puccinia purpurea* urediniospores collected from Hermitage Research Station, Warwick, Queensland (28.2102°S, 152.1041°E) in Queensland, Australia (White *et al*., 2015) were suspended in 100–150 mL distilled water with 1–2 drops of Tween 20, and sprayed onto plants using a Preval^®^ compressed air atomizer (Preval Sprayer Division). A complementary set of plants were treated identically but without urediniospores as controls. Inoculation chambers were closed to maintain high (80%+) humidity and placed in darkness at 20°C for 24 h before being transferred to diurnal conditions (16 h light, 8 h dark) to allow rust infection to develop.

### Anthracnose infection

Sorghum seeds were sown in rolls of germination paper and kept in darkness for 4 days at 28°C (Lo *et al*., 1996). Etiolated seedlings with elongated mesocotyls were inoculated with spore suspensions of *C. sublineolum*, at the concentration of ~3.0 × 10^6^ conidia/mL with gelatin as a wetting agent (0.25%). Inoculated plants were incubated at 100% relative humidity at room temperature for 24 h. Three independent infections were performed on elongated mesocotyls. The phenotypes were examined after 7 days following inoculation on an average of 35 plants per genotype. For each genotype the number of plants with mild symptom, strong symptom or no symptoms were recorded.

### Microscopy

At 6–8 dpi, fourth or fifth leaf samples were collected and submerged in 1M KOH and incubated for 48 h at 37°C with gentle agitation. The KOH solution was replaced with fresh 1M KOH solution after 18–24 h. The KOH solution was discarded, and the leaf material was washed gently 2–3 times with 50 mm Tris‐HCl, allowing material to incubate in the Tris‐HCl solution for 10–20 min per wash. 1–2 mL 50 mm Tris‐HCl and 10–20 μL 1 mg/mL wheat germ agglutinin conjugated to fluorescein isothiocyanate (WGA‐FITC, Sigma‐Aldrich, Castle Hill, NSW, Australia) were added, and samples incubated at ambient temperature for an hour or kept at 4°C before mounting on microscope slides. Stained leaf samples were mounted on slides using a few drops of 40% glycerol before covering with cover slips. GFP3 fluorescence filters were used on a Leica MZFLIII fluorescence dissecting microscope or a Zeiss Axioimager upright fluorescence microscope to score the presence of *P. purpurea* infection sites in the sampled leaves.

### Rust biomass assays

Chitin assays were carried out as described by Ayliffe *et al*. (2014). Three biological replicates of the 6th leaves were sampled 14 and 28 dpi and weighed. Leaves were cut into 1.5–2.0 cm fragments and submerged in 1M KOH with 0.15 Silwet L‐77 in Falcon tubes. Leaf samples are autoclaved at 121°C and 15 psi for 20 min, then washed gently three times in 50 mm Tris‐HCl pH 7.5. Plant samples were suspended in 50 mm Tris‐HCl pH 7.5 at the rate of 200 mg fresh weight per mL and homogenized by sonication for 1–2 min to form a fine uniform suspension. About 4 × 100 μL of each homogenate was transferred to 4 × 200 μL PCR tubes. About 10 μL of 1 mg/mL WGA‐FITC (Sigma‐Aldrich) was added to each homogenate in PCR tubes and left at ambient temperature for an hour. Homogenates were washed three times by centrifuging at 250 ***g*** for 3–5 min and carefully replacing the supernatants with 50 mm Tris‐HCl pH 7.5 using a micropipette. The final washed suspensions were transferred to a 96‐well fluorometer microtiter plate. Fluorescence values of each sample were measured in a Wallac Victor 1420 multilabel counter at 485 nm excitation and 535 nm emission wavelengths with a 1.0 s measurement time. Means of the technical replicate fluorescence values were calculated, and the standard errors were ascertained for biological replicates.

### Metabolite analysis

Samples of mesocotyl tissue (~200 mg each) from uninfected and *C. sublineolum* inoculated plants at 48, 72 and 96 hpi were collected, cut into segments, weighed and placed in acidified (0.1%) HPLC‐grade methanol. Metabolites were allowed to leach from the tissue at 4°C overnight. The composition of plant extracts was then determined by liquid chromatography‐tandem mass spectrometry (LC‐MS) in accordance with the protocols as described (Du *et al*., 2010; Lo *et al*., 1999). Authentic standards of luteolinidin, apigeninidin, luteolin and apigenin (Sigma) were used for metabolite identification and quantification.

### qRT‐PCR

The sixth leaf of plantlets were sampled at 0, 24 and 48 hpi, snap‐frozen in liquid nitrogen and stored at −80°C. RNA was isolated with the RNeasy^®^ Plant Mini Kit (QIAGEN, Chadstone Centre, VIC, Australia) according to manufacturer's instructions. About 1–2 μg RNA samples were subjected to first‐strand DNA synthesis in 20 μL reactions using Superscript^®^ III reverse transcriptase Life Technologies (Mulgrave, VIC, Australia). About 3 μL of 1‐in‐10 dilutions of first‐strand synthesis products were subjected to qPCR reactions using the C1000 Touch™ thermocycler with the CFX96™ Real‐Time System (Bio‐Rad, Gladesville, NSW, Australia). Reaction conditions included an initial denaturization at 95°C for 3 min; 40 cycles of denaturization at 95°C for 10 s and annealing/elongation at 60°C for 30 s, followed by a melt step range of 65–95°C with increments of 0.5°C. The sorghum *actin* gene (Pavli *et al*., 2011) was used as a reference gene for each qRT‐PCR experiment, and each qRT‐PCR experiment was repeated using the more stable sorghum reference gene *PP2A* (Reddy *et al*., 2016). qPCR primers specific for Lr34*res*,* SbL34* (Lr34 orthologue), *SbFNR*,* SbFNSII* and *SbDFR3* were used to measure the relative gene expressions at the different post‐inoculation time points are listed in Table 1. Experiments included three technical replicates of each of three respective biological replicates. Means of the ΔCq values were calculated, and Standard Errors were determined for the data. Gene expression values were log(base 2)‐transformed, and a repeated measures analysis was performed via the linear mixed model software asreml (Butler, 2009) in R (R Core Team, 2015). Means and SE bars in Figure 4 are back‐transformed to the scale of the raw expression levels.

**Table 1:** Primers used in QPCR gene expression analyses

Gene	Primer	Primer sequence (5′–3′)	Amplicon size (bp)	Reference
*SbActin*	Forward	CTAGCAGCATGAAGATCAAGGTG	134	Pavli *et al*. (2011)
	Reverse	GCCAGACTCGTCGTACTCAG		
*SbPP2A*	Forward	AACCCGCAAAACCCCAGACTA	138	Reddy *et al*. (2016)
	Reverse	TACAGGTCGGGCTCATGGAAC		
*Lr34res*	Forward	GGGAGCATTATTTTTTTCCATCA	242	This paper
	Reverse	ACTGGCAGAAGAACCTTGAAACA		
*SbL34* (Sb01g016775)	Forward	GGGAGCATTATATTTTTCCATCT	247	This paper
	Reverse	TAACTGGCAGAAGAACCTGGAAG		
*Flavone Synthase II* (*SbFNSII*, Sb02g000220)	Forward	CGCAAGACCACCGTCTTCTT	209	Du *et al*. (2010) This paper
	Reverse	GCCGGCACGGCCTGCATGGC		
*Dihydroflavonol 4‐reductase 3* (*SbDFR3*; Sb04g004290)	Forward	CGGATGTGACGATTGTTTGA	123	Liu *et al*. (2010)
	Reverse	GGGCATATTGGTTTGGAACTT		
*Flavanone 4‐reductase* (*SbFNR*; Sb06g029550)	Forward	GGGTAACAAGAAGACGATGAAGA	287	Kawahigashi *et al*. (2016)
	Reverse	CTGGATCCTGTGCCTCGAAGT		

### Reproductive yield components

Sorghum plants at the T2 generation and four replications were grown to physiological maturity, and intact panicles were harvested and dried at 37°C for 48 h. Individual panicles were weighed for each of the negative and positive Lr34 lines. Peduncle diameters were measured 1 mm above the last node using Vernier calipers. Kernels were separated from the panicles and 100 kernel quantities from transgenic and non‐transgenic lines were weighed.

## Conflict of interest

The authors declare no conflict of interest.

## Supporting information


**Figure S1** Genomic blot of EcoRV‐restricted DNA from transgenic Sorghum.
**Figure S2** Lr34 sib and transgenic sorghum penultimate leaves.
**Figure S3** Rust sporulation on sorghum leaves 5 and 6 at 15 days post‐inoculation with *P. purpurea*.
**Figure S4** (a) Pustule development and (b) fungal biomass on transgenic sorghum leaves one month post‐inoculation with *P. purpurea*.
**Figure S5** Rust sporulation at 14 days post‐inoculation in Lr34res transgenics and altered variants of Sb01g016775^−∆F525, Y613H^.
**Figure S6** Pathogen‐induced pigmentation 24–72 h post‐inoculation (a) Negative sib line. (b) Lr34‐2 single copy line. (c) Lr34‐5 3 copy line. (d) Lr34‐6 7 copy line.
**Figure S7** Relative gene expression of sorghum *Lr34* ortholog (*Sb01g016775*) at 0 and 24 h post‐inoculation.
**Figure S8** Flavonoid metabolites in sorghum mesocotyls after infection with *C. sublinoeleum*.
**Figure S9** Anthracnose symptoms (arrows and yellow bracket) following mesocotyl infection by *C. sublinoleum*.
**Figure S10** Comparison of peduncle diameters of transgenic sorghum lines. Data shown as mean ± SE from 4 to 6 biological replicates.
