# Supplementary material for: The wheat Lr34 multipathogen resistance gene confers resistance to anthracnose and rust in sorghum
Source: Plant Biotechnol J. 2017 Apr 20;15(11):1387–96. doi: 10.1111/pbi.12723 (PMC5633760; doi:10.1111/pbi.12723)
Supplement: Supplementary file 1 — Figure S1 Genomic blot of EcoRV‐restricted DNA from transgenic Sorghum. Figure S2 Lr34 sib and transgenic sorghum penultimate leaves. Figure S3 Rust sporulation on sorghum leaves 5 and 6 at 15 days post‐inoculation with P. purpurea. Figure S4 (a) Pustule development and (b) fungal biomass on transgenic sorghum leaves one month post‐inoculation with P. purpurea. Figure S5 Rust sporulation at 14 days post‐inoculation in Lr34res transgenics and altered variants of Sb01g016775−∆F525, Y613H. Figure S6 Pathogen‐induced pigmentation 24–72 h post‐inoculation (a) Negative sib line. (b) Lr34‐2 single copy line. (c) Lr34‐5 3 copy line. (d) Lr34‐6 7 copy line. Figure S7 Relative gene expression of sorghum Lr34 ortholog (Sb01g016775) at 0 and 24 h post‐inoculation. Figure S8 Flavonoid metabolites in sorghum mesocotyls after infection with C. sublinoeleum. Figure S9 Anthracnose symptoms (arrows and yellow bracket) following mesocotyl infection by C. sublinoleum. Figure S10 Comparison of peduncle diameters of transgenic sorghum lines. Data shown as mean ± SE from 4 to 6 biological replicates. [file PBI-15-1387-s001.docx]

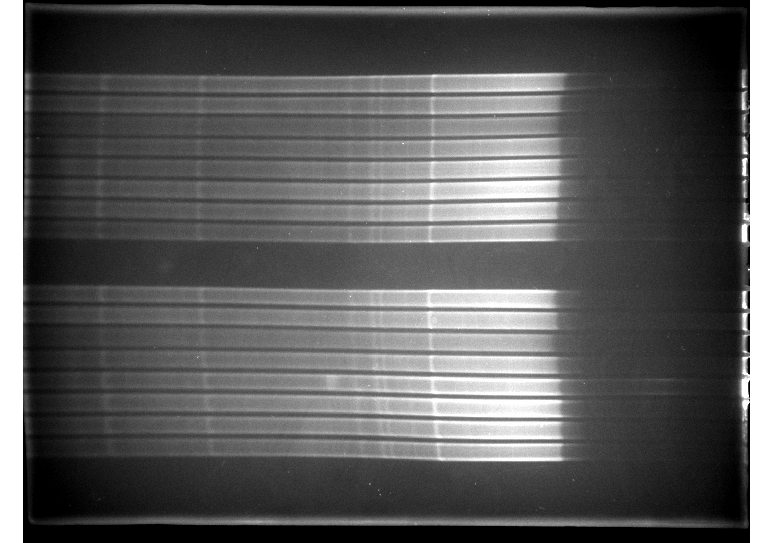

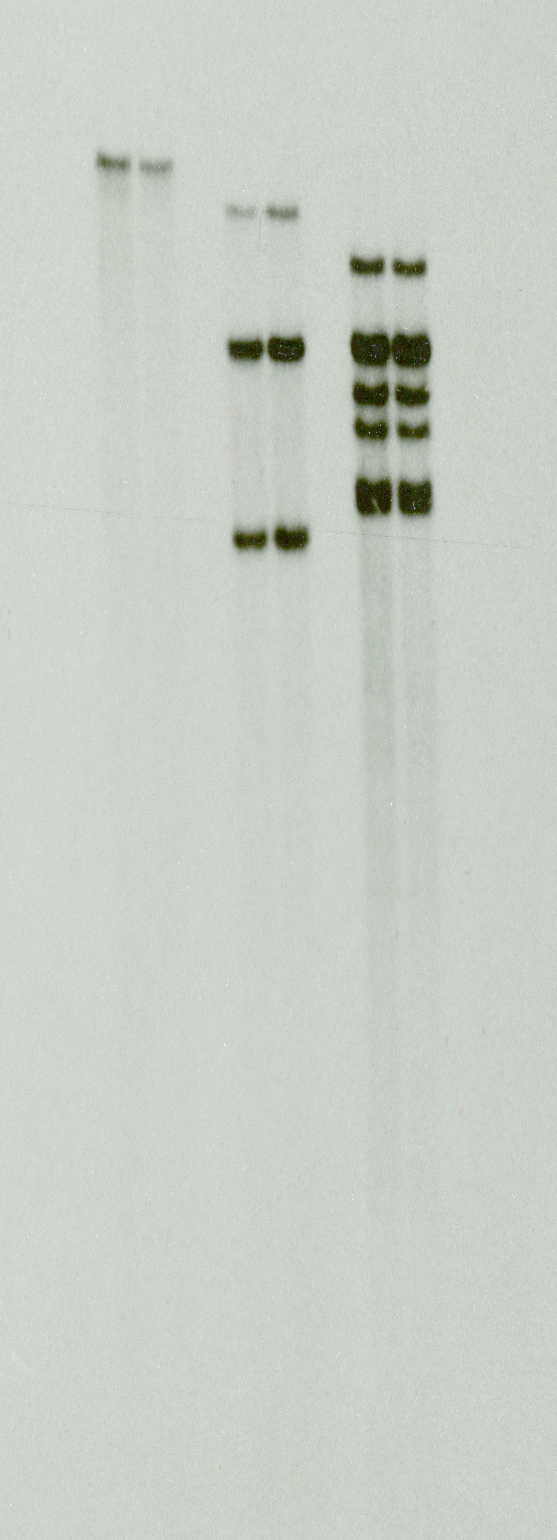


(b)

(a)

1 2 3 4 5 6 7 8

1 2 3 4 5 6 7 8

**Figure S1.** Genomic blot of EcoRV-restricted DNA from transgenic Sorghum. (a) 1% agarose gel and (b) subsequent Southern blot hybridised with Lr34*res* specific probe (Lr34-3’-UTR probe). Lanes 1 and 2. Lr34-2 single copy lines; Lane 3. Tx430 wild-type; Lanes 4 and 5. Lr34-5 multi-copy lines (3 gene copies); Lane 6. Lr34-5 negative sibling line; Lanes 7 and 8. Lr34-6 multi-copy lines (7 gene copies).


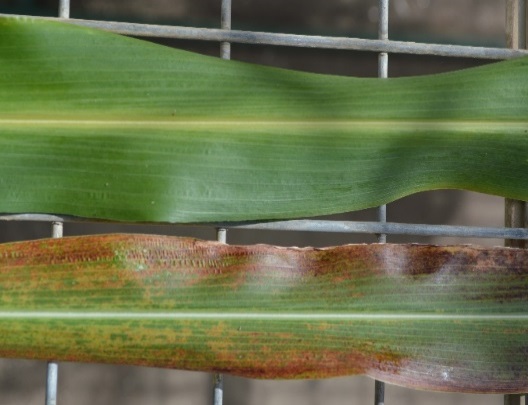

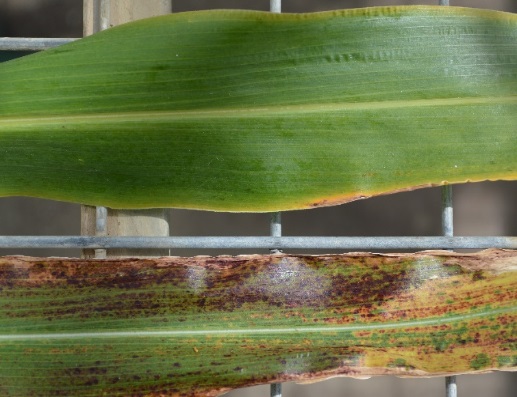

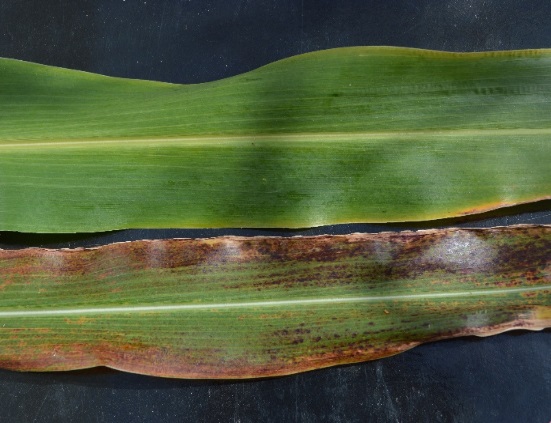

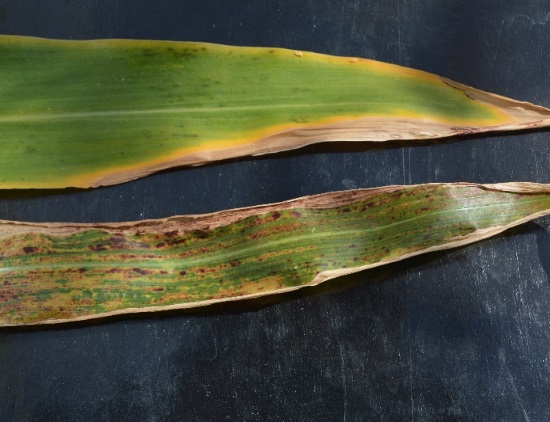


(a)

(b)

(c)

(d)

(e)

(f)

(g)

(h)

**Figure S2.** Lr34 sib and transgenic sorghum penultimate leaves. (a) Lr34-2 sib line without the transgene. (b) Lr34-5 (3 gene copy line). (c) Lr34-2 (single copy line). (d) Lr34-6 (7 gene copy line). (e and g) middle lamina and leaf tip respectively of Lr34-2. (f and h) middle lamina and leaf tip respectively of Lr34-6. Bronzing colour developed in the multicopy *Lr34res* transgenic adult plants at the booting stage of development.


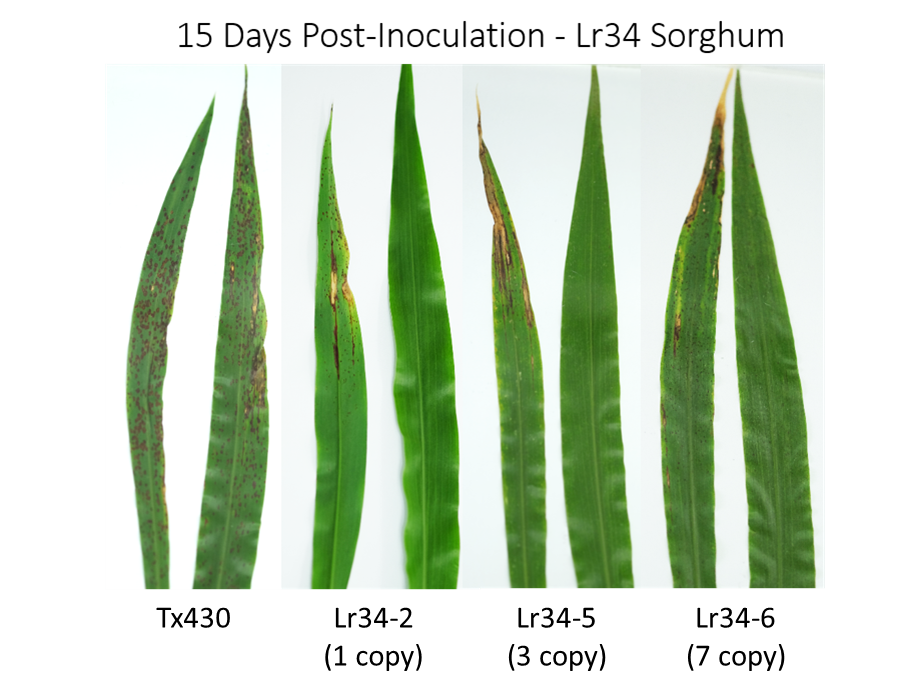


**Figure S3.** Rust sporulation on sorghum leaves 5 and 6 at 15 days post inoculation with *P. purpurea*.


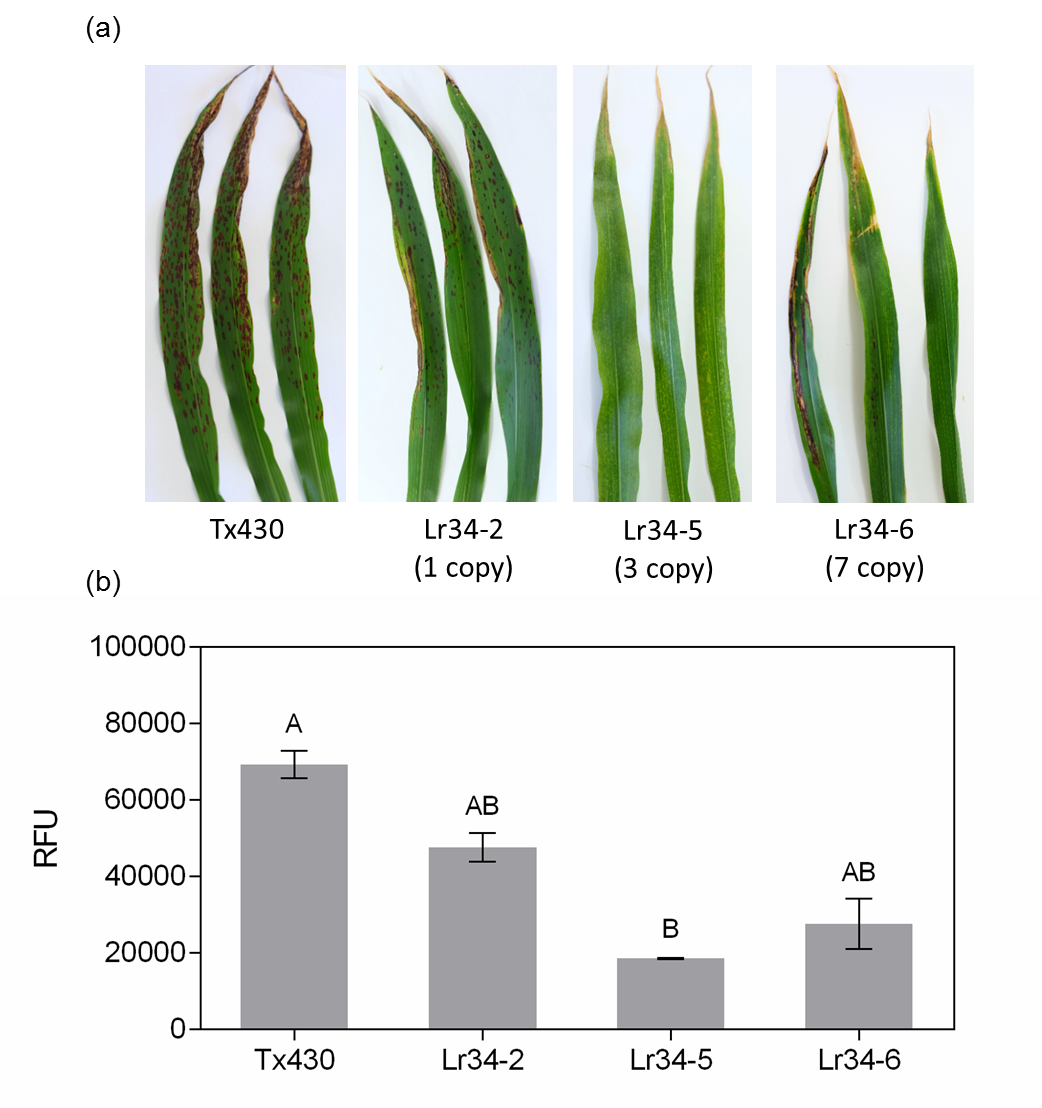


**Figure S4.** (a) Pustule development and (b) fungal biomass on transgenic sorghum leaves one month post-inoculation with *P. purpurea*.


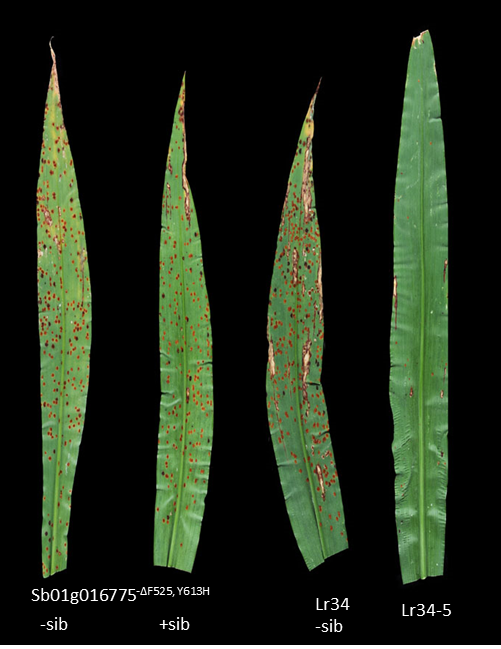


**Figure S5.** Rust sporulation at 14 days post inoculation in Lr34res transgenics and altered variants of Sb01g016775^-∆F525, Y613H^.


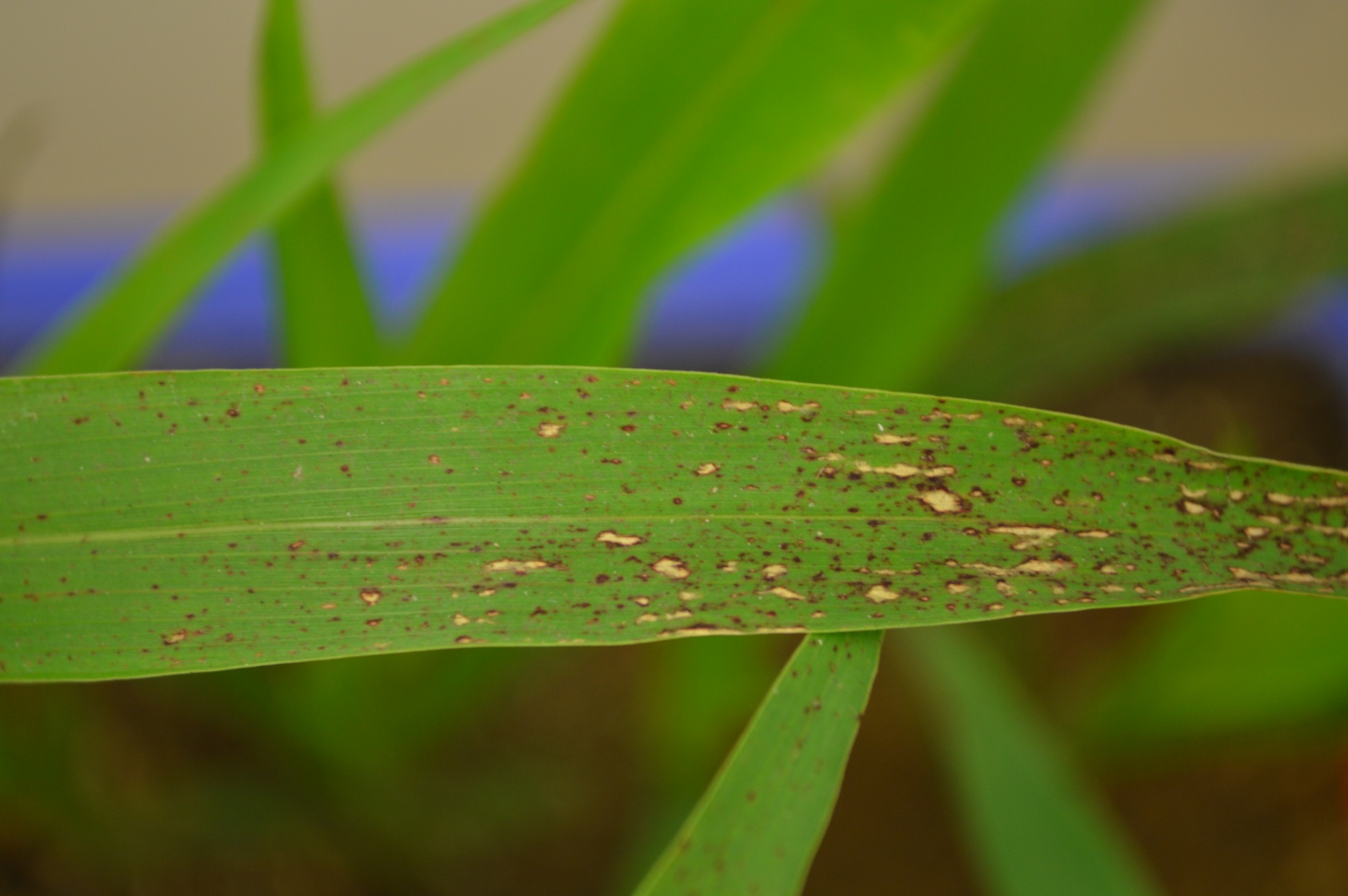

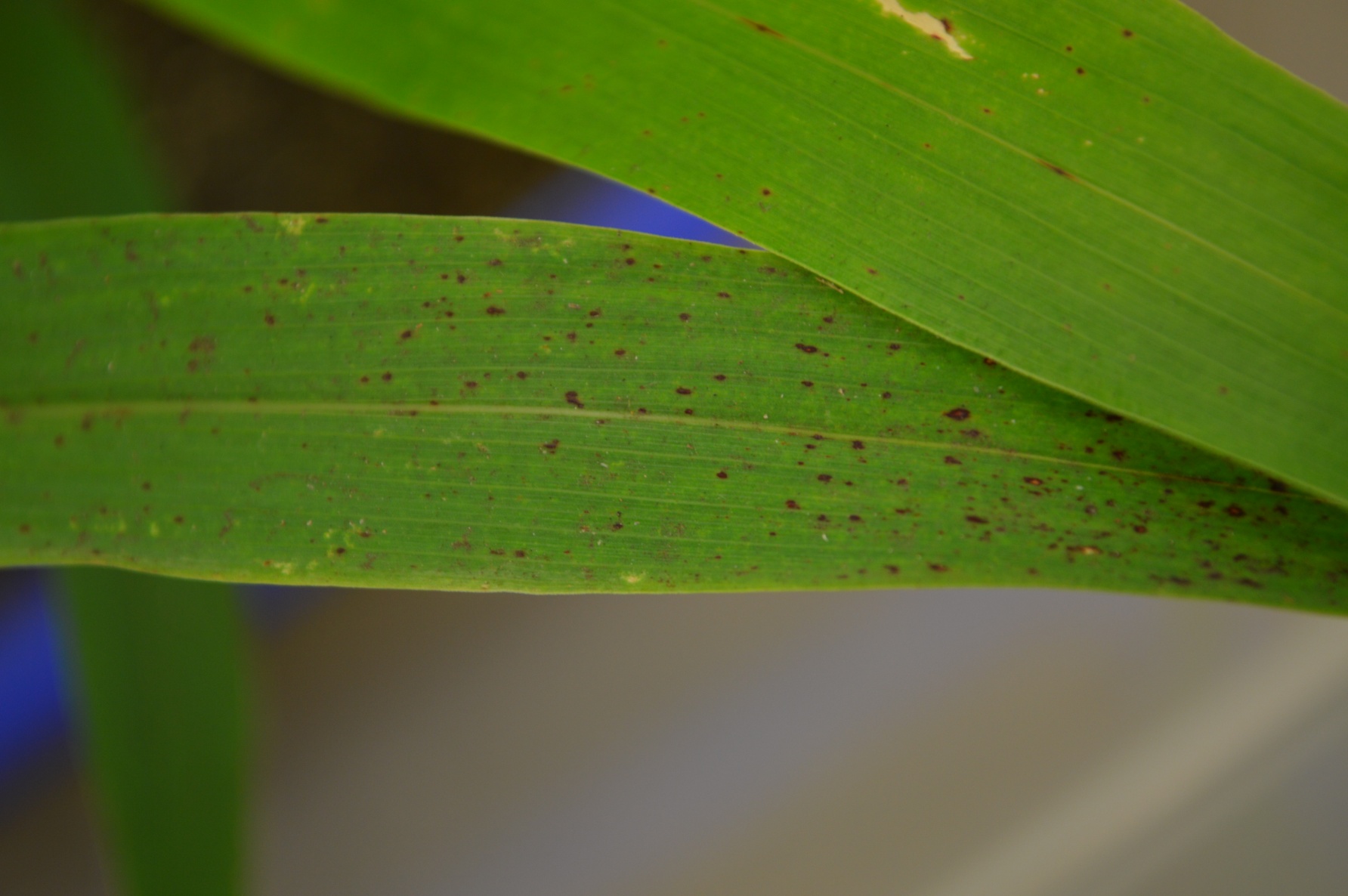

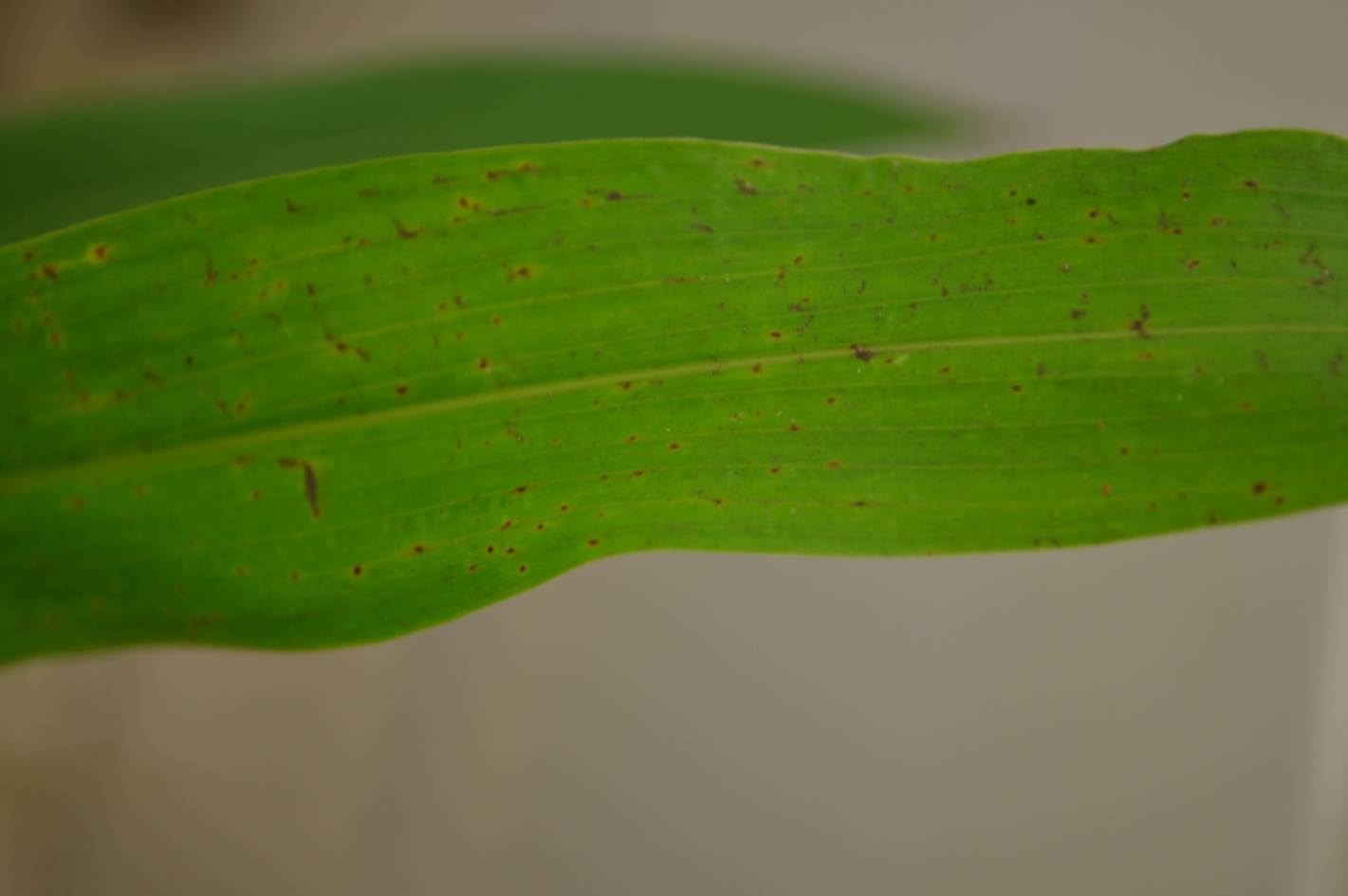

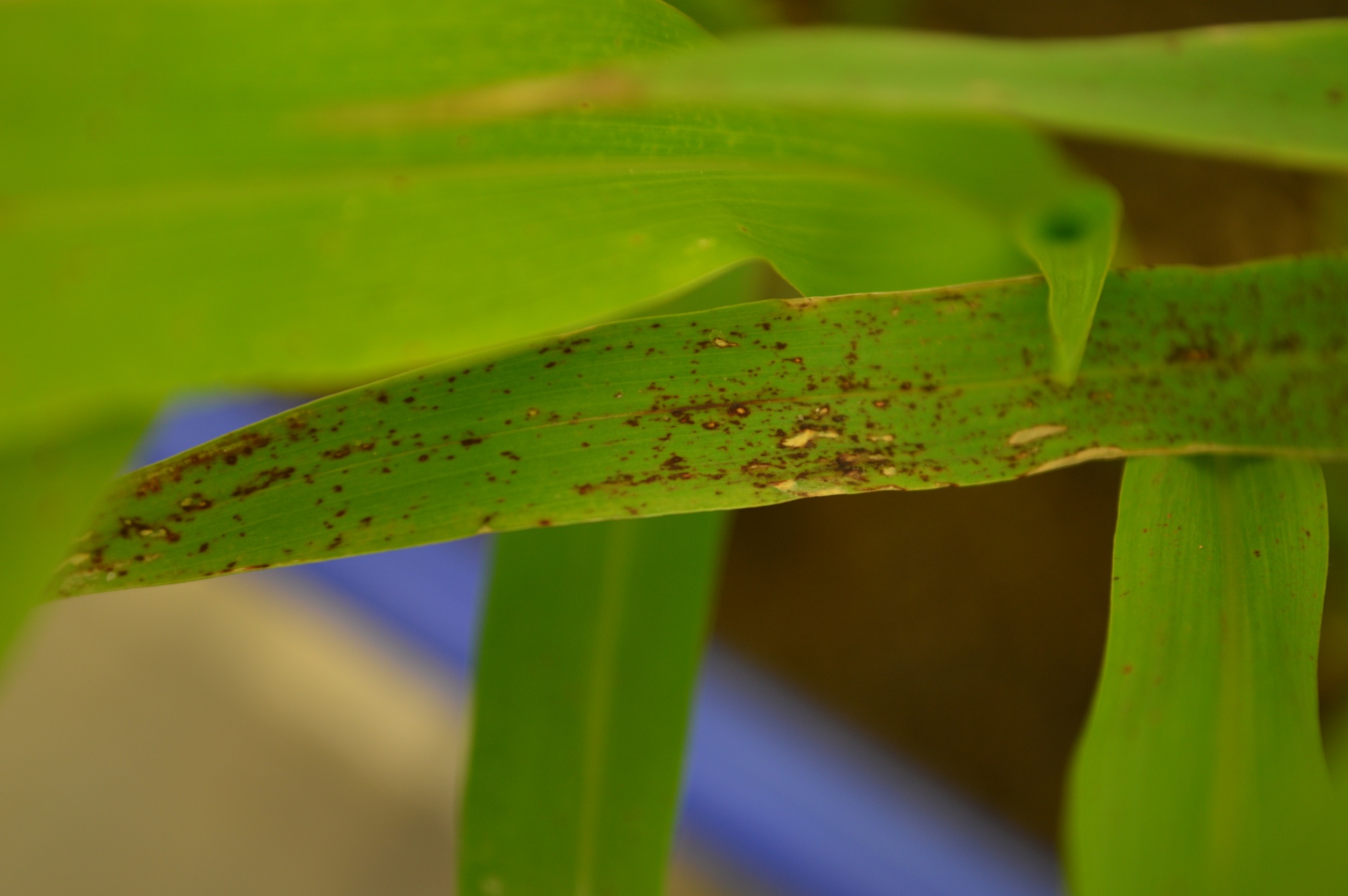


(a) -sib

(b) Lr34-2

(c) Lr34-5

(d) Lr34-6


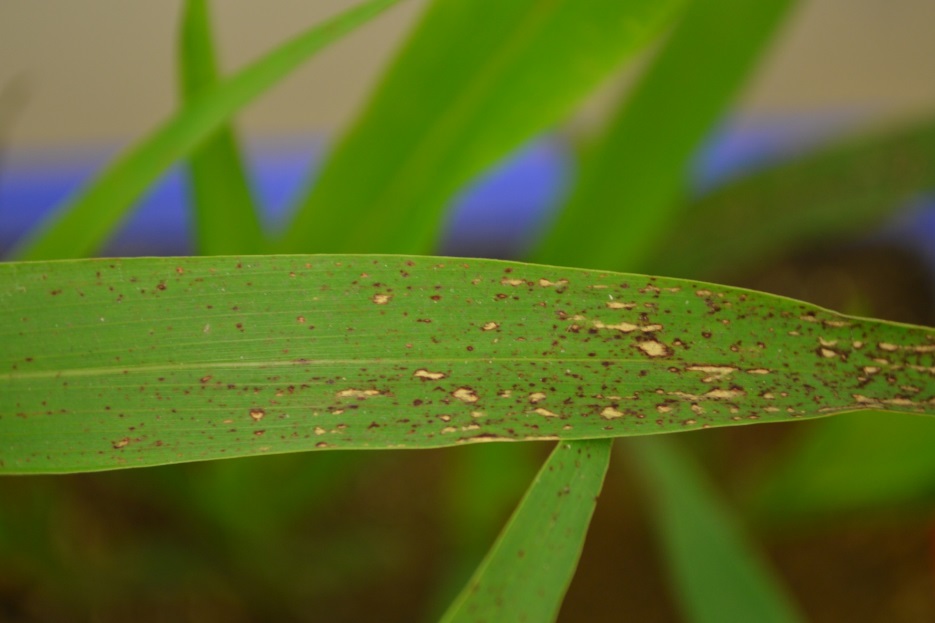

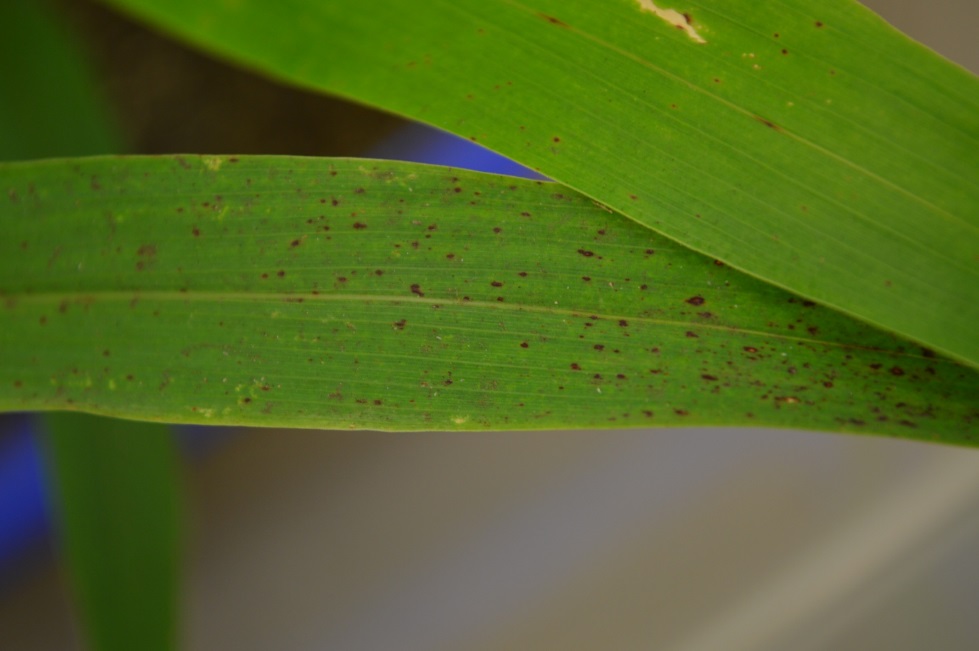

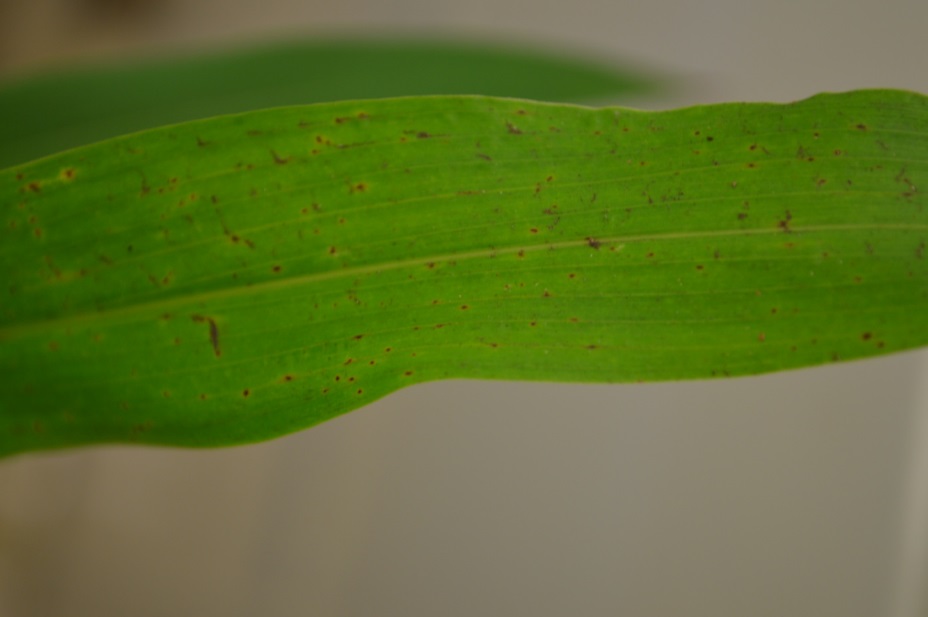

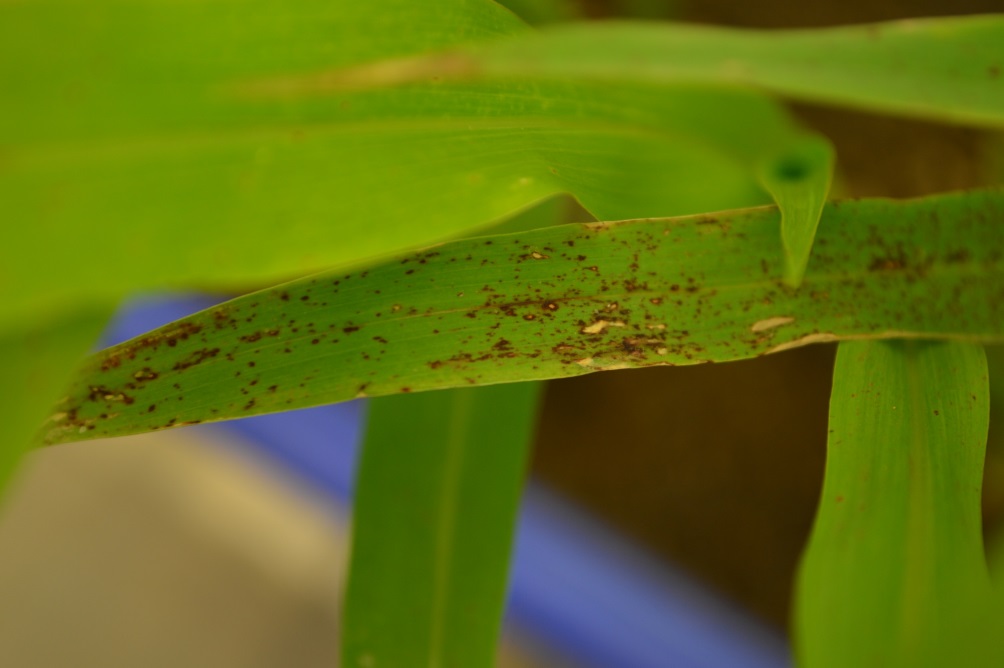


**#5-1 (0 copy)**

**#2-2 (1 copy)**

**#5-1 (3 copies)**

**#6-1 (3 copies)**

**Figure S6.** Pathogen induced pigmentation 24-72 hours post inoculation (a) Negative sib line. (b) Lr34-2 single copy line. (c) Lr34-5 3 copy line. (d) Lr34-6 7 copy line.


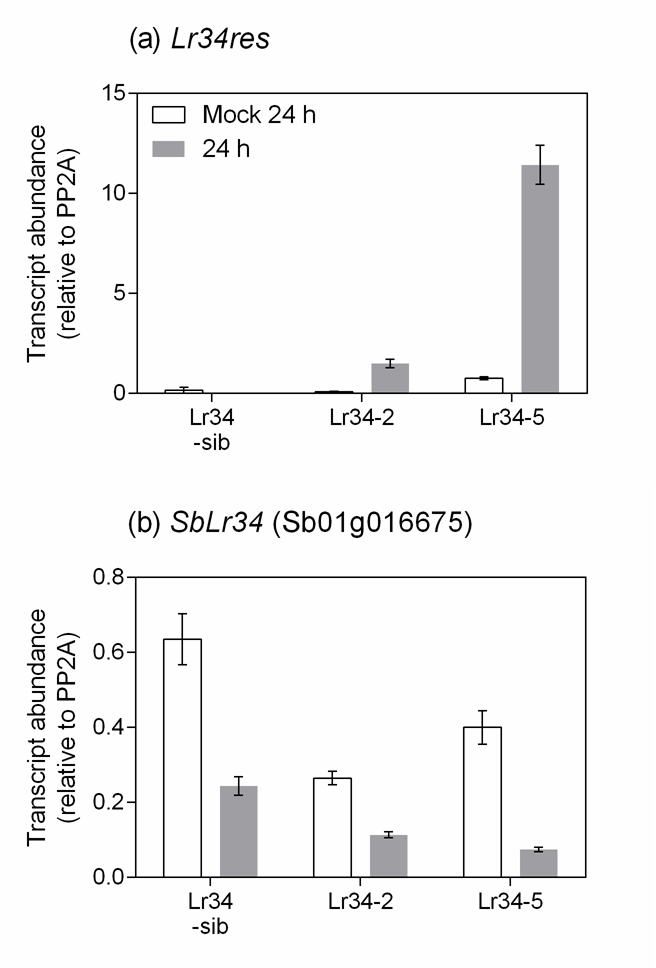


**Figure S7.** Relative gene expression of sorghum *Lr34* ortholog (*Sb01g016775*) at 0 and 24 hours post-inoculation.

**
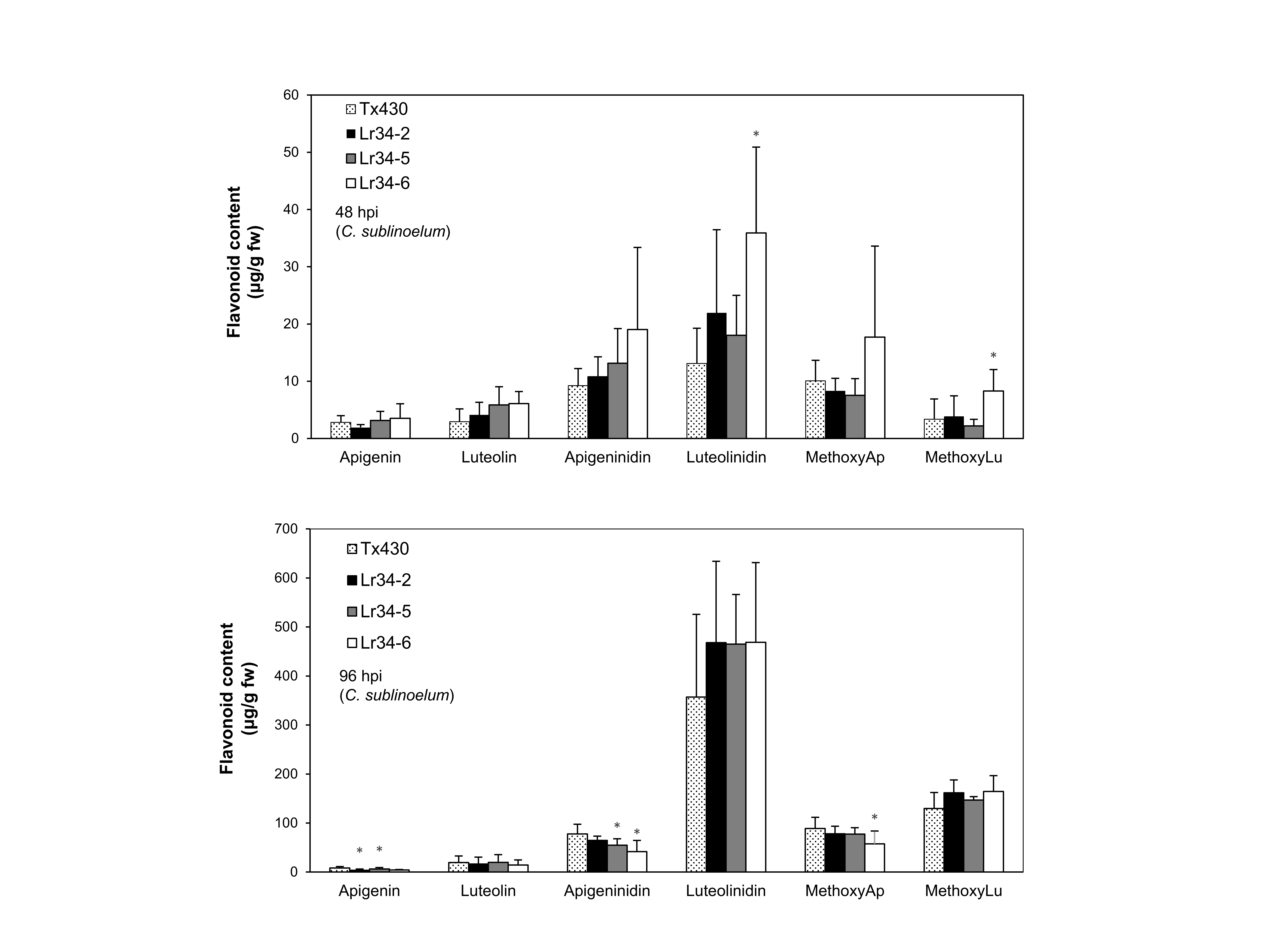
**

**Figure S8.** Flavonoid metabolites in sorghum mesocotyls after infection with *C. sublinoeleum.* (a) 48 hpi. (b) 96 hpi. *p<0.05 (*t*-test).


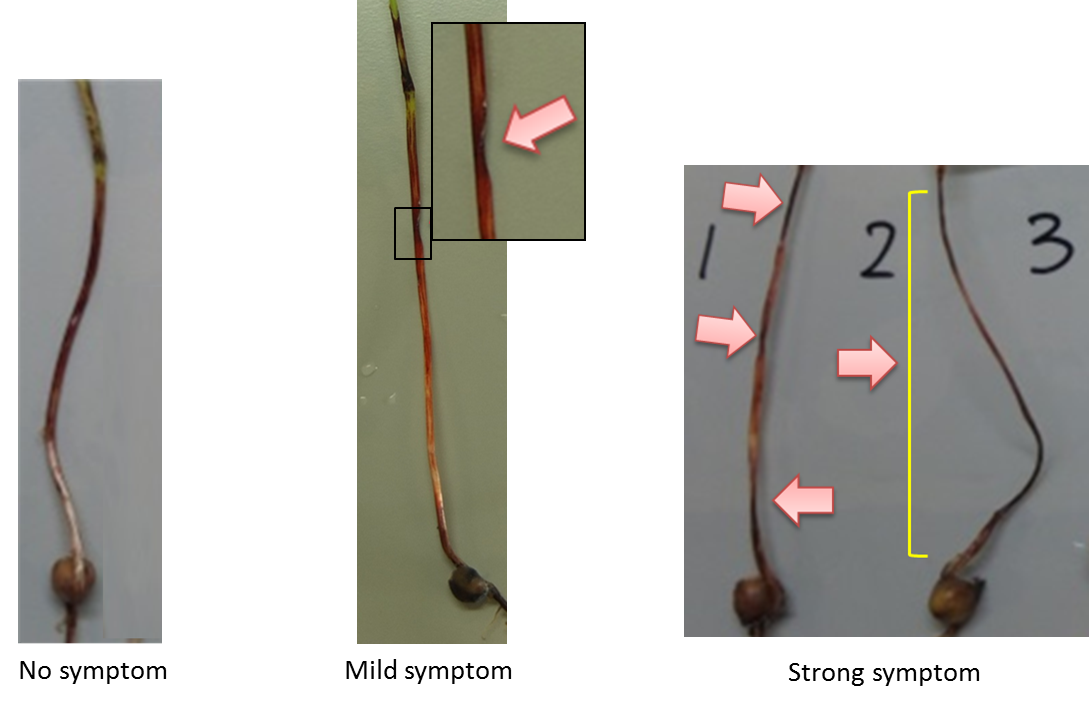


**Figure S9.** Anthracnose symptoms (arrows and yellow bracket) following mesocotyl infection by *C. sublinoleum*.





**Figure S10.** Comparison of peduncle diameters of transgenic sorghum lines. Data shown as mean ± SE from 4-6 biological replicates.
